# Unraveling SREBF1’s role in elevating colorectal cancer prognosis through proliferation and migration inhibition

**DOI:** 10.1371/journal.pone.0327503

**Published:** 2025-07-16

**Authors:** Dongling Li, Qinrui Cai, Fan Xu, Ling Lin, Xiaoya Zhou, Li Li, Yao Chen, Tianlin Feng, Yuanxiu Gan, Chenhua Zhang, Fan Yang

**Affiliations:** 1 Central laboratory of Chongqing Emergency Medical Center, Chongqing University Central Hospital, School of Medicine, Chongqing University, Chongqing, China; 2 College of Pharmacy and Bioengineering, Chongqing University of Technology, Chongqing, China; 3 Department of Critical Care Medicine of Chongqing Emergency Medical Center, Chongqing University Central Hospital, School of Medicine, Chongqing University, Chongqing, China; 4 Chongqing Key Laboratory of Emergency Medicine of Chongqing Emergency Medical Center, Chongqing University Central Hospital, School of Medicine, Chongqing University, Chongqing, China; 5 School of Mechanical, Electrical and Information Engineering, Putian University, Putian, Fujian, China; Karpagam University: Karpagam Academy of Higher Education, INDIA

## Abstract

Sterol Regulatory Element-Binding Protein 1 (SREBF1), a central regulator of lipid metabolism, has unclear pan-cancer roles and clinical implications. This study integrated various databases and functional experiments to systematically investigate the heterogeneous characteristics of SREBF1 across cancers. Pan-cancer analysis revealed significant upregulation of SREBF1 in multiple cancer types, including colorectal cancer (CRC). Survival analysis demonstrated that SREBF1 overexpression serves as an independent risk factor for poor prognosis in colorectal cancer patients. Focusing on CRC, functional studies revealed that SREBF1 drives tumor progression by enhancing cancer cell proliferation and migration, while its knockdown induces cell cycle arrest and apoptosis in HCT116 cells. Mechanistically, SREBF1 is implicated in lipid metabolic reprogramming and interacts with the tumor immune microenvironment, also with genetic alterations. This study highlights the regulatory role of SREBF1 in pan-cancer contexts and provides novel insights into its potential as a prognostic biomarker and therapeutic target, particularly in colorectal cancer.

## 1. Introduction

Cancer is a disease marked by the abnormal growth of cells, which often spreading to nearby tissues. It can occur in any part of the body due to genetic factors, environmental influences, or lifestyle choices [1,2]. And pan-cancer analysis reveals commonalities and variations in human malignancies by studying molecular abnormalities across different cancer types [3]. Utilizing resources like TCGA and GEO, this analysis aids in developing combination and personalized therapies for diverse cancer models [4,5].

Sterol Regulatory Element-Binding Transcription Factor 1 (SREBF1) is a specific isoform of the Sterol Regulatory Element-Binding Transcription Factor (SREBF) family [6]. It is also known as SREBP-1 or SREBP-1a. SREBF1 plays a crucial role in regulating the expression of genes involved in the biosynthesis of fatty acids, triglycerides, and cholesterol. It is highly expressed in tissues involved in regulating of lipid metabolism, including liver, adipose tissue, and muscle [7,8]. SREBF1 is activated in response to low cellular levels of lipids, which leads to the upregulation of genes involved in lipogenesis and lipid uptake, CERS6 and Lipin-1 are transcriptionally activated by SREBF1 [9,10]. This process is critical for maintaining energy homeostasis and preventing metabolic disorders. Additionally, SREBF1 has been linked a number of other biological processes, including inflammation, cell proliferation, and differentiation. The dysregulation of SREBF1 activity has been associated with a number of pathological conditions, including cardiovascular disease, certain types of cancer, and non-alcoholic fatty liver disease. [11,12]. Studies have suggested that SREBF1 may have a role in regulating immune function. One of how SREBF1 could modulate immune function is through its regulation of lipid metabolism, as lipids and cholesterol play an important role in immune cell function, SREBF1 reprograms metabolism by promoting ROS production, decreasing GSH levels, and reducing mitochondrial membrane potential, thus protecting prostate cancer cells from ferroptosis [13]. But the precise role of SREBF1 in other cancer immune function is still being elucidated.

The rapid proliferation of cancer cells can be supported by an increase in fatty acid synthesis [14]. Furthermore, alterations in lipid metabolism have been observed to stimulate tumor growth, immune evasion, cancer recurrence and metastasis [15]. Thus we hypothesize that SREBF1 may influence the progression of cancers through certain metabolic or immunological pathways. In this study, we discovered that high expression of SREBF1 was positively correlated with poor prognosis in colorectal cancer, and significantly inhibited the proliferation and migration of colorectal cancer cells after SREBF1 was knocked down. Furthermore, we have also conducted further analyses across multiple cancer types. The analysis encompasses differential gene expression, protein correlation, pathway, and prognostic analysis of various tumor types and stages. Additionally, the study sought to ascertain the association between SREBF1 expression and the presence of immune infiltrating cells. All the findings indicated that SREBF1 could be a valuable prognostic biomarker in many types of cancers as a novel therapeutic strategy.

## 2. Materials and methods

### 2.1. Gene expression analysis

The TIMER2.0 database (http://timer.cistrome.org/) was employed to investigate differential expression levels of SREBF1 between pan-cancer tumor tissues and matched adjacent normal tissues [16]. Subsequently, the GEPIA2.0 database (http://gepia2.cancer-pku.cn/#index) was utilized to evaluate the correlation between patients’ pathological stages and SREBF1 expression across all TCGA cancer cohorts [17]. For proteomic analysis, the CPTAC (Clinical Proteomic Tumor Analysis Consortium) module within the UALCAN database (http://ualcan.path.uab.edu/index.html) was applied to examine protein-level expression patterns of SREBF1 across diverse cancer types [18,19]. Additionally, the Human Protein Atlas (HPA) database (https://www.proteinatlas.org) was accessed to validate SREBF1 expression levels in tumor tissues relative to their corresponding normal tissues through multi-omics data integration [20].

### 2.2. Genetic alteration analysis

The cBioPortal database (https://www.cbioportal.org/) was utilized to gather the data about the alteration frequency, mutation type, mutation site information, and threedimensional (3D) structure of SREBF1 in all TCGA databases [21]. The promoter methylation levels of SREBF1 in various types of cancers were analyzed by the UALCAN database.

### 2.3. The infiltration of immune cells

We analyzed the association between SREBF1 and the immune infiltration levels in different cancer types by using the TIMER2.0 (http://timer.cistrome.org/), and we used Pearson’s correlation coefficient to calculate the correlation between the SREBF1 and various immune cell types, including CD4 + T cells, CD8 + T cells, neutrophils, macrophages, eosinophils, and natural killer cells.

### 2.4. Single-cell sequencing

CancerSEA database (http://biocc.hrbmu.edu.cn/CancerSEA/) as a professional single cell sequencing, we utilized it to analyze the differential expression of SREBF1 in different cell subpopulations, then generated the results of RB (Retinoblastoma), HNSCC (Head and Neck Squamous Cell Carcinoma) and ODG (Oligodendroglioma) by T-SNE diagram [21]. We used the Singerbox (http://www.sangerbox.com/) to obtain the heatmap between the SREBF1 and the immune correlation.

### 2.5. Survival analysis

Using the GEPIA2 analysis to assess the relationship between the expression of SREBF1 and the prognostic of patients, including overall survival (OS), and the patients divided into the low expression and high expression groups based on the median value. The hazard ratio was calculated based on the Cox PH Model.

### 2.6. Gene enrichment analysis

The GEPIA2.0 database (http://gepia2.cancer-pku.cn/#index) included the TCGA and GTEx data, which could generate the top 100 SREBF1-related genes, and then we conducted the heatmap between the top six related genes and SREBF1. Gene ontology (GO) and Kyoto Encyclopedia of Genes and Genomes (KEGG) enrichment analyses were employed to analyze the SREBF1-influenced biological functions and signaling pathways.

### 2.7. Cell lines

SW480, HCT116, NCM460, LS174T, HT29, and LOVO were bought from Cell Resource Center, Peking Union Medical College (PCRC). SW480, HCT116 were cultured in IMDM supplemented with 10% fetal bovine serum and 1% penicillin/streptomycin, while the NCM460, LS174T, HT29, and LOVO were cultured in DMEM supplemented with 10% fetal bovine serum and 1% penicillin/streptomycin. All cell lines were authenticated by short tandem repeat (STR) profiling and tested as mycoplasma-free.

### 2.8. Quantitative PCR (qPCR)

Total RNA was extracted using Steady Quick RNA Extraction Kit (ACCURATE BIOLOGY, AG21023), then used ABScript III RT Master Mix for qPCR with gDNA Remover (ABclonal, RK20429) reverse transcription into cDNA by following the protocol. RT-qPCR was conducted using 2X Universal SYBR Green Fast qPCR Mix (ABclonal, RK21203), according to the manufacturer’s instructions. Beta-Actin was used to normalize the expression of the target genes. Primer sequences for RT-qPCR were as follows: primer for SREBF1 (forward primer: CGGAACCATCTTGGCAACAGT, reverse primer: CGCTTCTCAATGGCGTTGT) and primer for Beta-Actin (forward primer: CATGTACGTTGCTATCCAGGC, reverse primer: CTCCTTAATGTCACGCACGAT).

### 2.9. Western blot

The cells were washed with the 1X iced-PBS and lysed in RIPA cell lysis buffer for 5 minutes, then ultrasonic the mixed buffer for 10 min, and centrifugation at 14,000 × g, 4 °C for 15 min, the supernatant was subsequently subjected to protein analysis. The protein lysates were separated on SDS-PAGE gels and transferred onto a 0.45 mm PVDF membrane for 2 hours. The membrane was then subjected to overnight incubation at 4 °C with SREBF1 antibodies (Proteintech). The next day, after three washes in TBST, the membrane was incubated with the appropriate secondary antibody for 1 hour at room temperature. Finally, the membrane was exposed by the VILBER image-forming system.

### 2.10. Cell growth assay

A total of 2000 cells were seeded in 96-well plates and cultured for 96 hours. Thereafter, 10 μL of CCK-8 solution was added to each well daily. After incubation for 60 minutes at 37°C, the optical density (OD) values at 450 nm were determined using a microplate reader (Tecan, Austria) and normalised to the corresponding control.

### 2.11. Colony formation assay

In total, 1000 cells were plated in 6-well plates and cultured for approximately 14 days. Cell colonies were fixed with 4% formaldehyde (P0099, Beyotime) and stained with 0.1% crystal violet (C8470, Solarbio) for 15 minutes, and the colonies were photographed and counted manually.

### 2.12. Transwell assay

Migration assays were performed using Transwell chambers in the presence of Matrigel (Corning 354,480). SREBF1 knockdown HCT116 cells (1 × 10^5^ cells/well) were seeded in the upper chamber with serum-free medium, and 700 µl of 10% FBS was added to the lower chamber of the 24-well plate. After 48 hours of incubation, the cells on the upper surface of the filter were completely removed and washed by PBS. The filters were then fixed in 4% paraformaldehyde and stained with crystal violet (Beyotime C0121).

### 2.13. Patients and tissues

Human CRC tissues and the corresponding adjacent non-tumor tissues were obtained from the Affiliated Central Hospital of Chongqing University, Chongqing Emergency Medical Center, Chongqing, China (2024 Lunshen No. (52)). All participants provided written informed consent, and the study was approved by the local ethics committees. The studies were conducted in accordance with the Declaration of Helsinki and the International Ethical Guidelines for Biomedical Research Involving Human Subjects (CIOMS).

### 2.14. Statistical analysis

Statistical analysis was conducted using one-way ANOVA to compare SREBF1 expression across public datasets. Chi-squared tests were used to assess differences in clinical data and immune checkpoint inhibitor responses between subgroups. Survival outcomes differences were evaluated using the Kaplan-Meier method with log-rank testing. Hazard ratios were calculated via univariate and multivariate Cox regression. For normally distributed data, Student’s t-tests or ANOVA were used to analyze SREBF1 mRNA levels and results from CCK-8, cell cycle, and Annexin FITC & PI assays across groups. Image analysis was performed with ImageJ software. Significance was set at p < 0.05, with adjustments made using the Benjamini-Hochberg method. Graphpad Prism 10.1.2 were used to conduct statistical analysis.

## 3. Results

### 3.1. The expression of SREBF1 in human pan-cancer

**Fig 1:**
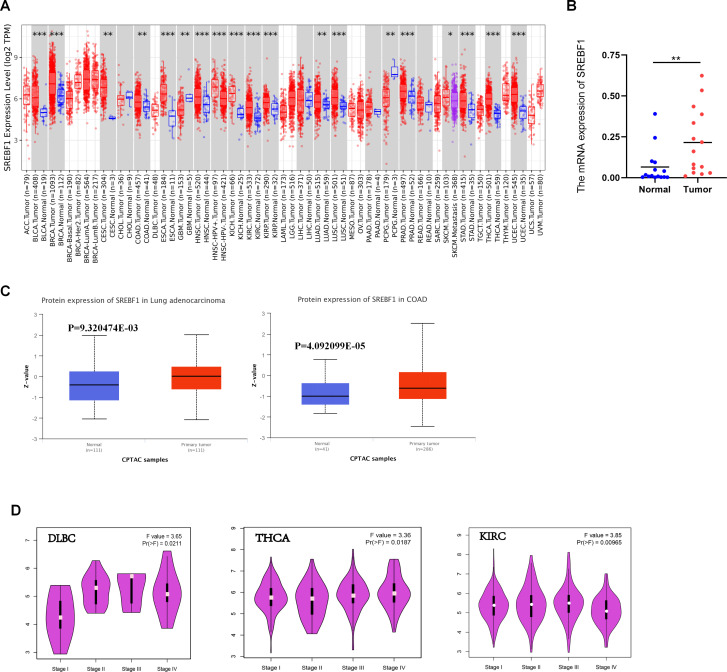
The expression of SREBF1 in pan-cancer. (A) SREBF1 expression in different cancers from TIMER2.0. *p < 0.05; **p < 0.01; ***p < 0.001. (B) RT-PCR result of SREBF1 expression in Colorectal cancer and adjacent normal tissue. (C) The protein levels of SREBF1 in LUAD and COAD were analyzed. (D) SREBF1 expression levels and the pathological stages were analyzed.

To better understand the effect of SREBF1, we initially evaluated the level of SREBF1 expression in different cancers. As Fig 1A showed, compared the expression between tumor and adjacent non-tumor tissues, SREBF1 expression was significantly up-regulated in Bladder Urothelial Carcinoma(BLCA), Breast Cancer (BRCA), Cervical Squamous Cell Carcinoma (CESC), Colon Adenocarcinoma (COAD), Esophageal Squamous Cell Carcinoma (ESCA), Glioblastoma (GBM), Head and Neck Squamous Cell Carcinoma (HNSC), Kidney Chromophobe (KICH), Kidney Renal Clear Cell Carcinoma (KIRC), Kidney Renal Papillary Cell Carcinoma (KIRP), Lung Adenocarcinoma (LUAD), Lung Squamous Cell Carcinoma (LUSC), Prostate Adenocarcinoma (PRAD), Skin Cutaneous Melanoma (SKCM), Stomach Adenocarcinoma (STAD), Thyroid Carcinoma (THCA), Uterine Corpus Endometrial Carcinoma (UCEC). Only in GBM and Pheochromocytoma and Paraganglioma (PCPG), adjacent normal tissues have higher expression of SREBF1. RT-PCR analysis of clinical specimens also demonstrated that the expression level of SREBF1 was significantly higher in colorectal cancer tissues compared to paired adjacent normal tissues (Fig 1B). Then, the protein levels of SREBF1 in pan-cancer tissues were further analyzed. The following results demonstrated that the expression levels of the total SREBF1 protein were elevated in primary tissues of Colon Cancer (COAD), and LUAD compared with normal tissues (Fig 1C). Besides, we explored the connection between SREBF1 expression levels and tumor stages, which led us to discover a significant effect of SREBF1 expression on the stages of the patients with Diffuse Large B-Cell Lymphoma (DLBC), KIRC, and THCA (Fig 1D).

### 3.2. Prognostic assessment value of SREBF1 in pan-cancer

**Fig 2:**
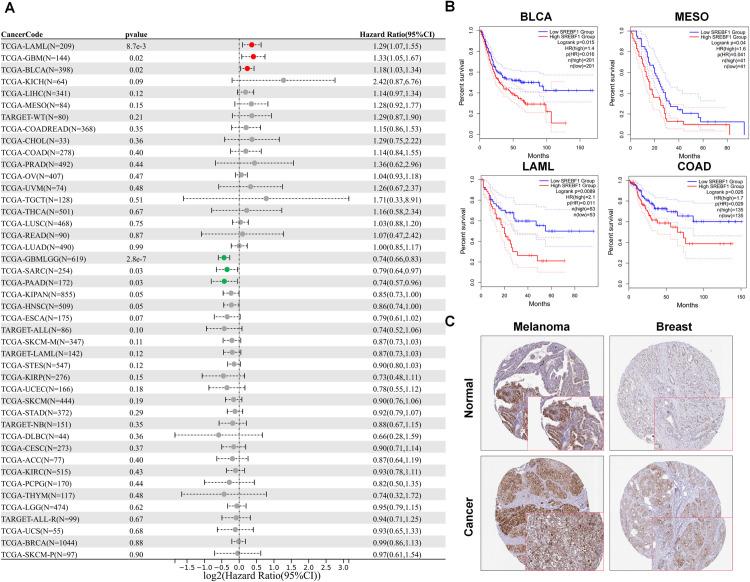
Prognostic values of SREBF1 expression in pan-cancer. (A) Forest plot of the correlation between SREBF1 and overall survival (OS) in patients with tumors. (B) We used the GEPIA2 tool to investigate the impact of SREBF1 gene expression on patients’ prognosis, including overall survival (OS) with BLCA, MESO and LAML, and disease-free survival (DFS) with COAD. (C) HPA platforms displayed the upregulated expression of SREBF1 in tumor tissue derived from melanoma and breast.

We initially assessed the prognostic value of SREBF1 in pan-cancer OS. SREBF1 emerged as a risk factor in LAML, GBM, and BLCA, it also exhibited a favorable protective effect in GBMLGG, SARC, and PAAD (Fig 2A). Next, we assessed the prognostic value of SREBF1 in pan-cancer OS and DFS. We found that high expression of SREBF1 associated with poor OS in BLCA (p = 0.016), Mesothelioma (MESO) (p = 0.041), Acute Myeloid Leukemia (LAML) (p = 0.011), and patients with high SREBF1 expression in COAD exhibited significantly inferior disease-free survival (DFS) (p = 0.029) (Fig 2B and S1A Fig). Considering the significant role of SREBF1 expression in predicting outcomes for COAD, we concentrated our efforts on investigating the biological functions of SREBF1 in COAD. The clinical and genetic characteristics of COAD patients from the TCGA cohort are detailed in **Table 1**. Notable differences were identified in lymphatic invasion, gender, and weight (median with interquartile range).

**Table 1:** Clinical characteristics of SREBF1 -high and -low expressed COAD patients.

Characteristics	Low expression of *SREBF1* (n = 239)	High expression of *SREBF1* (n = 239)	*P* value
Lymphatic invasion, n (%)			0.005
No	149 (34.3%)	117 (27%)	
Yes	71 (16.4%)	97 (22.4%)	
Gender, n (%)			0.044
Female	102 (21.3%)	124 (25.9%)	
Male	137 (28.7%)	115 (24.1%)	
Weight, median (IQR)	83.65 (68.367, 97.425)	75 (63, 91.1)	0.033

Additionally, we verified the expression of SREBF1 by analyzing the IHC results sourced from the HPA database. Our analysis suggested that SREBF1 was predominantly strongly or positively expressed in tumor tissue originating from breast, and melanoma (Fig 2C). Overall, the findings imply that SREBF1 has the ability to serve as a prognostic marker in multiple types of cancer.

### 3.3. Investigation of genetic alterations of SREBF1 in Pan-cancer

**Fig 3:**
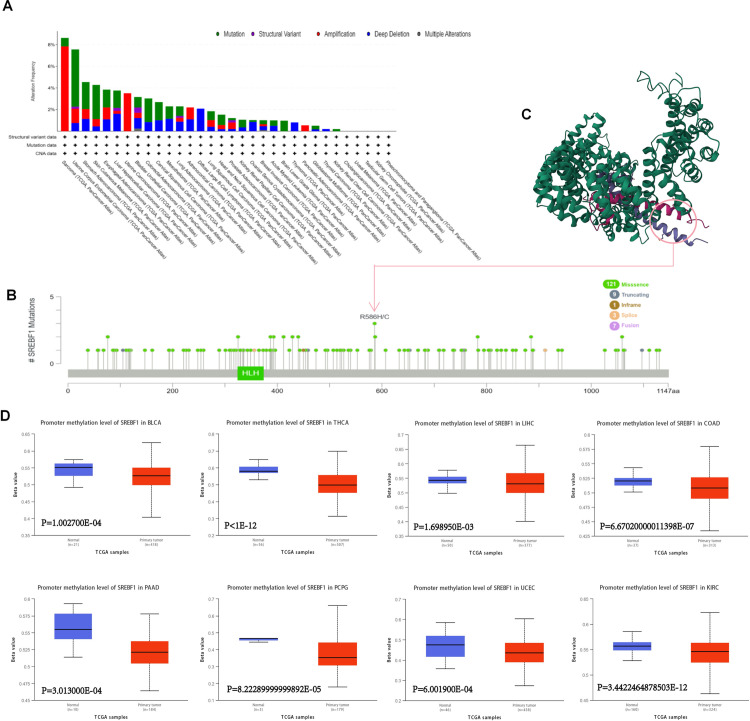
SREBF1 gene mutation in various cancers. (A, B) The alteration frequency of different mutation types (A) and mutation site (B) of SREBF1 in pan-cancer. (C) SREBF1 mutation site was shown in the 3D protein structure of SREBF1. (D)The methylation values of SREBF1 between normal and primary tumor tissues were analyzed using UALCAN tool.

We explored the gene mutations of SREBF1 in various cancers. As shown in Fig 3A, the most common DNA change was mutation. Patients with sarcoma (SAR), UCEC, STAD, SKCM, ESCA, Liver Hepatocellular Carcinoma (LHC), BLCA, COAD, CESC, MESO, LUAD, LUSC, HNSC, PRAD, KIRP, OV, BRCA, AML, Brain Lower Grade Glioma (LGG), GBM, and KIRC carried SREBF1 mutation. Additionally, the analysis results reveled the high SREBF1 amplification in SARC and UCS (>2%) (Fig 3A). The sites, types, and case numbers of the SREBF1 gene modification were further displayed, showing that the misssence was the main mutation type of SREBF1 (Fig 3B). We also observed the R586H/C site in the 3D model of the SREBF1 protein (Fig 3C). By analyzing genetic mutation data, researchers can uncover the relationship between genes and cancer, explore the molecular mechanisms of cancer occurrence and development, and provide significant reference value for subsequent drug research [22].

DNA methylation is closely related to cancer occurrence and progression [23,24]. We investigated the DNA methylation of SREBF1 by the UALCAN. A marked decrease of SREBF1methylation level was observed in BLCA, THCA, CESC, LIHC, COAD, KIRC, PCPG, UCEC, and PAAD tissues compared to normal tissues by the UALCAN database. The methylation level of SREBF1 in HNSC, GBM, ESCA, BRCA, LUSC, and PRAD was increased (Fig 3D and S1B Fig).

### 3.4. Correlation between the immune infiltration cells and SREBF1 in COAD

Immune cells play an important role in the tumor microenvironment (TME), and plenty of evidence indicated that cancer cells could interact with various components of the TME, then promote immune evasion and ultimately drive tumor growth, recurrence, and metastasis [25,26]. To identify new targets and biomarkers of the cancer which can help develop more targeted and personalized immunotherapies that can effectively attack cancer cells while minimizing harm to healthy cells. So in this section, we conducted the analysis of the correlation between immune infiltration cells and SREBF1 specifically in COAD.

**Fig 4:**
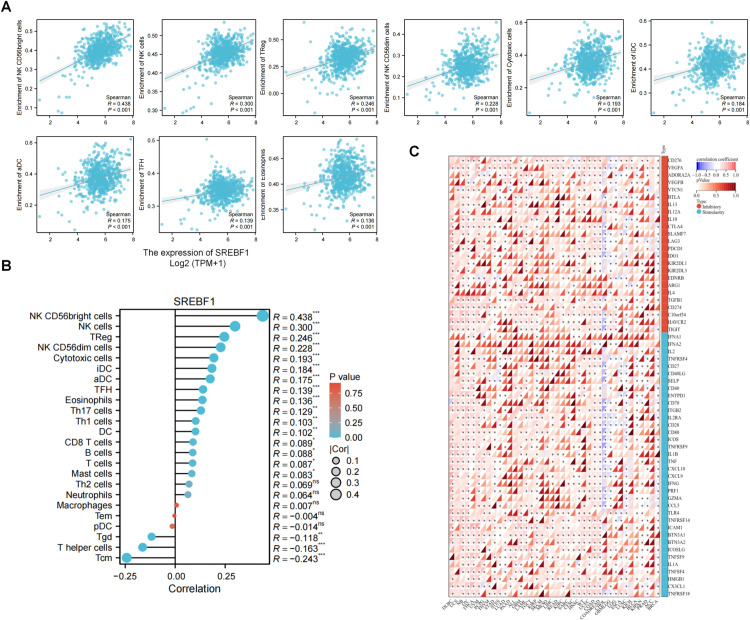
The correlation between SREBF1 and COAD immune microenvironment. (A) The correlation between SREBF1 and various immune cell types in COAD. ns > 0.05, *p < 0:05, **p < 0:01, ***p < 0:001. (B) The association of SREBF1 with different immune cells in COAD. (C) Correlation analyses of the SREBF1 expression with immune checkpoint genes in pan-cancer. Positive correlation (0–1) are indicated with the red color, while negative correlation (−1 to 0) are indicated with the blue color. p-value < 0.05 is considered as statistically significant. A cross indicates non-significant correlations.

We conducted a analysis to evaluate the relationship between SREBF1 expression levels and the presence of various immunocytes. Our findings indicated a significant positive correlation between SREBF1 expression and the increased presence of several immunocyte types. Notably, we found robust positive relationships with NK CD56bright cells, NK cells, Treg cells, NK CD56dim cells, cytotoxic cells, and T cells, among others, as illustrated in (Fig 4A). Moreover, the application of the ssGSEA algorithm to assess immunocyte signatures further confirmed the positive association between SREBF1 and a total of 18 different immunocyte types (Fig 4B). Immune checkpoints play a crucial role as key constituents in the clinical immunotherapy for tumors. The association between the SREBF1 expression and checkpoints in pan-cancer was also evaluated. And the finding demonstrated a strong correlation between elevated levels of immune checkpoints and the SREBF1 in ACC, notably KIR2DL3, IL2RA, CD80, IFNG, BTN3A2, and IL1A, also IFNA1 wass significantly associated with SREBF1 in the majority of cancers (Fig 4C).

### 3.5. The expression of SREBF1 at single-cell levels

**Fig 5:**
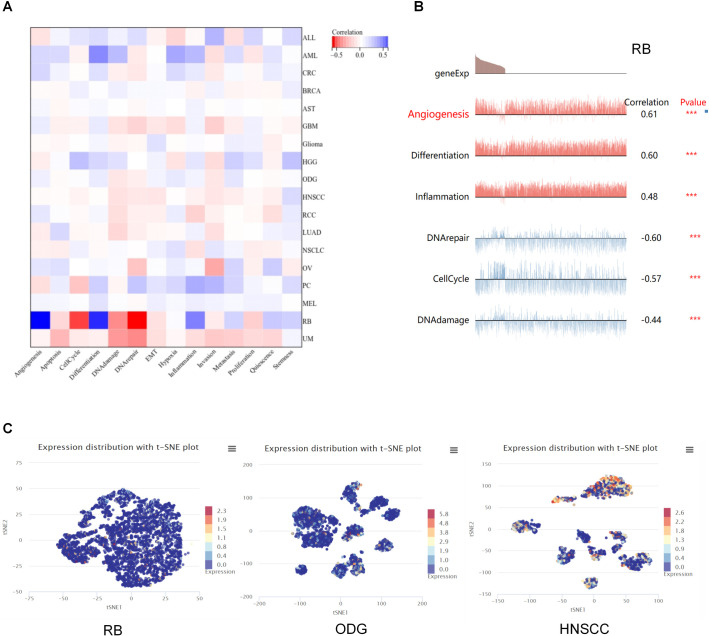
The expression levels of SREBF1 at single-cell levels. (A, B) The relationship between SREBF1 expression and different functional states in tumors was explored by the CancerSEA tool. *p < 0.05; **p < 0.01; ***p < 0.001. (C) SREBF1 expression profiles were shown at single cells from RB, ODG and HNSCC diagram.

Single-cell transcriptome sequencing is a molecular biology technique that allows for the analysis of gene expression at the level of individual cells. In RB, SREBF1 expression was positively related to most tumor biological behaviors such as DNA repair, DNA damage, invasion, apoptosis, and metastasis (Fig 5A). Also, SREBF1 expression in RB was found to be positively correlated with angiogenesis and differentiation (Fig 5B). In addition, SREBF1 expression profiles were shown at single-cell levels from RB, ODG, and through the T-SNE diagram, it can be observed from Fig 5C that there are distinct SREBF1 + cellular subsets and SREBF- subsets in HNSCC. Further functional annotation can elucidate the impact of SREBF1 expression on the functions of tumor cell subsets, such as proliferation and migration.

### 3.6. Functional enrichment analysis of SREBF1 in cancers

**Fig 6:**
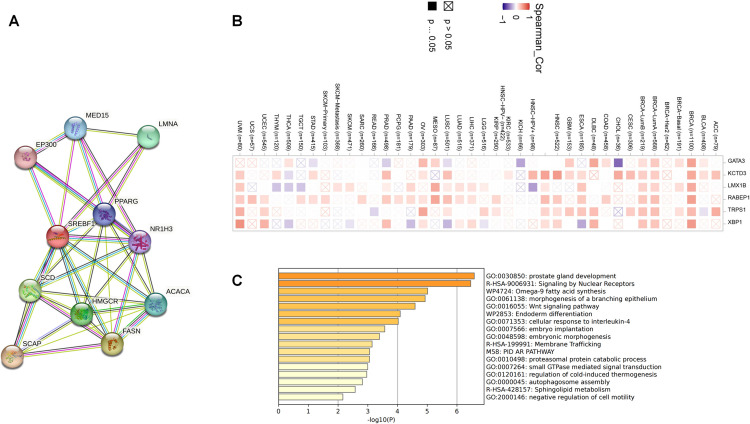
Functional enrichment analysis of SREBF1-related genes. (A) SREBF1-related genes were obtained from the BioGRID web tool, and 15 proteins were displayed. (B) The heatmap confirmed that SREBF1 expression was positively correlated with the six genes (GATA3, KCTD3, LMX1B, RABEP1, TRPS1, and XBP1) in pan-cancer. (C) GO and KEGG enrichment analyses of SREBF1-related pathway.

To gain deeper insights into the molecular mechanism of the SREBF1 gene in tumors, we conducted a functional enrichment analysis on SREBF1-associated genes that exhibit expression correlation with SREBF1. By utilizing the STRING tool, we identify 10 binding proteins that have been experimentally validated to interact with SREBF1. Subsequently, a protein-protein interaction (PPI) network was then constructed for these proteins to further elucidate their relationship with SREBF1 (Fig 6A). We also by utilizing the GEPIA2.0 tool obtained the top 100 genes associated with SREBF1 expression in pan-cancer. Among these genes, X-box binding protein 1 (XBP1), potassium channel tetramerization domain containing 3 (KCTD3), rabaptin, RAB GTPase binding effector protein 1 (RABEP1), transcriptional repressor GATA binding 1 (TRPS1), GATA binding protein 3 (GATA3), LIM homeobox transcription factor 1 beta (LMX1B) observed strong interactions with SREBF1 (Fig 6B). We also performed enrichment analysis using KEGG and GO, and the results indicated that SREBF1 might potentially participate in multiple pathways, including prostate gland development, signaling by nuclear receptors, and omega-9 fatty acid synthesis (Fig 6C). In summary, SREBF1 primarily functions in pathways closely related to cellular metabolism and hormone synthesis.

### 3.7. SREBF1 knockdown inhibit cell proliferation and migration

**Fig 7:**
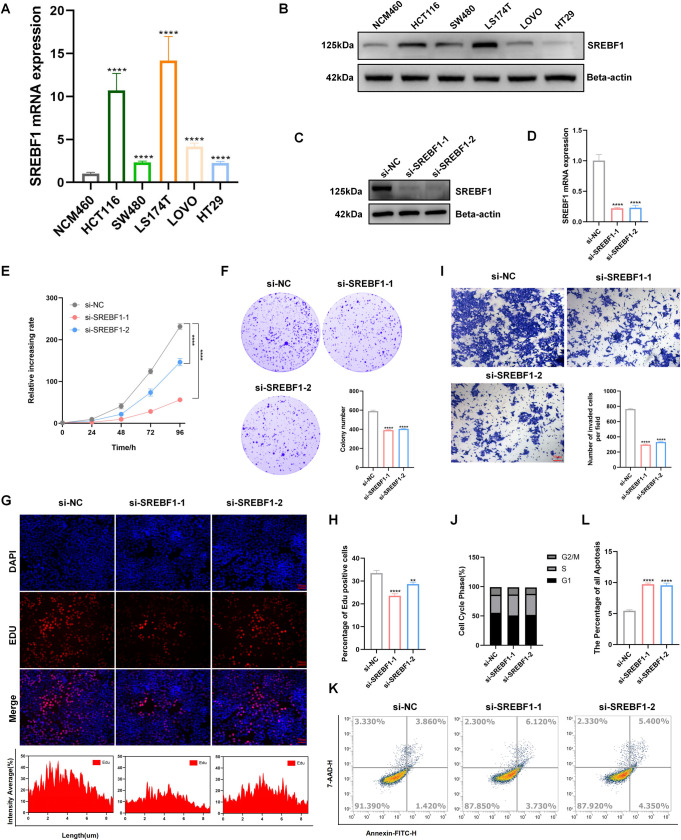
SREBF1 knockdown inhibits the proliferation and migration of HCT116 cell. (A) RT-PCR result of SREBF1 expression in human colorectal normal and tumor cell lines. (B) Western blotting of SREBF1 differential expression in human colorectal normal and tumor cell lines. (C) Western blotting of SREBF1 knockdown in protein level. (D) RT-PCR shows SREBF1 knockdown in mRNA level. (E) Cell proliferation assay by CCK8 (n ≥ 3). (F) Colony assay (n ≥ 3). (G-H) EDU assay and statistical analysis (n ≥ 3). (I) Transwell assay (n ≥ 3). (J) Cell cycle analysis by FlowCytometry (n ≥ 3). (K) Apoptosis analysis by FlowCytometry (n ≥ 3). ** means p < 0.01; *** means p < 0.001, **** means p < 0.0001 .

We verified the expression of SREBF1 in human colorectal cell lines, including both normal and tumor cells. Compared to the normal colorectal cell line NCM460, we identified a significantly increase in both mRNA and protein levels of SREBF1 in human colorectal cancer cell lines, particularly in the HCT116 and LS174T cell lines (Fig 7A,7B). To further validate the function of SREBF1 in tumor proliferation and migration, we selected the colorectal cancer cell line HCT116 to study the effect of SREBF1 knockdown in vivo (Fig 7C,7D). The colony, CCK8, and EDU results indicated SREBF1 knockdown significantly inhibited HCT116 cell proliferation, and the transwell also showed that indicated SREBF1 knockdown significantly inhibited HCT116 cell migration (Fig 7E–7H). The flow cytometry results demonstrated that SREBF1 knockdown could induce cell cycle arrest, with a decrease in G1 phase and an increase in S phase (Fig 7J). Moreover, SREBF1 knockdown could significantly induce cell apoptosis (Fig 7K,7L).

## 4. Discussion

In earlier studies, SREBF-1 was shown to play a key role in lipid and glucose metabolism, resulting in changes in cell growth, survival and invasiveness. In our research, we comprehensively analysed SREBF1 in several cancers in our research. The expression of SREBF1 was significantly increased in most types of cancers, only in GBM and PCPG with lower expression levels. Patients with high expression level of SREBF1 have been found to have a worse prognosis, such as BLCA, MESO, LAML, and COAD. Moreover, we discovered the evident impact of SREBF1 expression on the patients’ stages in DLBC, KIRA, and THCA. Mature SREBF1 primarily participates in fatty acid and triglyceride metabolism [27]. In various human cancers, signaling pathway is activated, then promotes the transcriptional activation of SREBP1, thereby facilitating intracellular lipid synthesis in tumor cells [28]. Notably, our analysis revealed a body weight-associated disparity in SREBF1 protein expression across cancer types. Specifically, LUAD, GBM, and ccRCC exhibited significant SREBF1 expression differences between normal and weight groups (S2 Fig). As a master transcriptional regulator of lipid metabolism and cholesterol biosynthesis, SREBF1 may be modulated through obesity-altered metabolic pathways, potentially mediating weight-dependent cancer progression. These findings suggest that weight management may have an important impact on the treatment and prognosis of certain cancers.

Although few studies have demonstrated that the overexpression of SREBF1 is closely related to many types of tumors, in HCC, the overexpression of SREBF1 is considered to be one of the major factors causing the proliferation and metastasis of cancer cells [29]. In breast cancer, high expression levels of SREBF1 are closely related to adverse outcomes and chemotherapy resistance, its specific mechanism is still not fully understood and requires further research [30]. Based on our research results, we discovered that SREBF1 is significantly overexpressed in BRCA, and over time, the DFS period for patients with high SREBF1 expression is notably lower than for those with lower expression levels. Our research found that the significant overexpression of SREBF1 and its association with poor prognosis in CRC, we further confirmed through cell experiments that SREBF1 promotes the proliferation and migration of colorectal cancer cells.

Genetic mutations and amplification have multifaceted impacts in cancers, such as genetic mutations can lead to alterations in intracellular signaling pathways, affecting cell growth and division; gene amplification may result in the overexpression of oncogenes, promoting the occurrence and progression of tumors [31]. In this research, we have found SREBF1 genetic alterations, such as mutations and amplification in different forms of cancers. These genetic alterations may affect the tumor’s response to treatment, providing potential targets for cancer therapy. Moreover, there are significant differences in the level of SREBF1 methylation between normal tissues and tumor tissues. In addition, Studies have demonstrated that SREBF1 forms a reciprocal regulatory loop with TP63/KLF5 to promote survival and migration in squamous cell carcinoma [32]. Sustained PI3K-AKT-mTOR signaling activation further enhances SREBP1-mediated lipid biosynthesis, conferring ferroptosis resistance in cancer cells [33]. Notably, nobiletin suppresses tumor growth by inhibiting SREBP1 nuclear translocation, deactivation of the PI3K/Akt/mTOR pathway, and induction of autophagy-dependent cell death [34]. These findings collectively establish SREBF1 as a central regulator of lipid metabolism and cell fate determination. Consistent with these mechanisms, our GO and KEGG enrichment analysis reveals significant involvement in omega-9 fatty acid synthesis, sphingolipid metabolism and signaling pathway. Importantly, we identified previously underappreciated associations with immune response pathways and developmental differentiation processes, expanding the known functional of SREBF1 in oncogenesis.

Infiltrating immune cells impact pan-cancer outcomes via dynamic interactions with the tumor microenvironment, influencing tumor progression, immune evasion, and therapeutic response [35]. Our pan-cancer analysis revealed that SREBF1 expression exhibits tumor-type-specific correlations with immune infiltration. In COAD, elevated SREBF1 levels showed significant positive correlation with T helper cell infiltration, while displaying negative association with NK CD56bright cells (Fig 6A–B). This regulation suggests SREBF1 may simultaneously enhance adaptive immunity while suppressing innate immune surveillance. The Th cell correlation aligns with SREBF1’s potential role in lipid-mediated antigen presentation, as omega-9 fatty acids are crucial for dendritic cell maturation. Conversely, the NK cell suppression might stem from sphingolipid-mediated inhibition of NKG2D receptor signaling, a mechanism warranting experimental validation. Notably, the strong association between SREBF1 and PD-L1 expression in COAD (Fig 6C) implies its involvement in immune checkpoint regulation. This creates therapeutic rationale for combining SREBF1 inhibitors (fatostatin [36]) with anti-PD-1/PD-L1 agents in colorectal cancers. Currently, our understanding of the functions of SREBF1 in the human immune system is limited. There exists a research gap regarding the roles of SREBF1 in the tumor immune microenvironment, and our findings indicate that focusing on SREBF1 could be a hopeful strategy for immunotherapy.

Despite the comprehensive analysis of SREBF1 in colorectal cancer, there are inevitable limitations in this study. First, the exact mechanisms by which SREBF1 influences tumor proliferation and immune evasion remain to be fully elucidated. Second, further clinical trials are required to clarify the therapeutic potential of targeting SREBF1 in colorectal cancer.

In summary, our comprehensive analysis of SREBF1 in colorectal cancer reveals that its expression is significantly associated with clinical outcomes, lipid metabolism, and immune cell infiltration. These findings provide valuable insights into the multifaceted role of SREBF1 in tumorigenesis and highlight its potential as a therapeutic target in colorectal cancer.

## 5. Conclusion

We systematically analyzed the characteristics of the SREBF1 from many aspects of pan-cancer, including expression, survival, prognosis, genetic mutation, and immune cell infiltration. And further identified the gene function in colorectal cancer cell growth and migration. The findings indicated that SREBF1 has the potential to be a new prognostic and immune-related biomarker for cancer patients. The study sheds light on the various roles of SREBF1 in different types of cancer and reveals new perspectives on the potential influence of the SREBF1 in pan-cancer.

## Supporting information

S1 Fig (A) We used the GEPIA2 tool to investigate the impact of SREBF1 gene expression on patients’ prognosis with disease-free survival (DFS). (B) The methylation values of SREBF1 between normal and primary tumor tissues.
(TIF)

S2 Fig SREBF1 expression level of protein in LUAD, HNSCC, Clear cell RCC and GBM with different weight. (TIF)
