# Supplementary figures and images for: Unraveling SREBF1’s role in elevating colorectal cancer prognosis through proliferation and migration inhibition
Source: PLoS One. 2025 Jul 16;20(7):e0327503. doi: 10.1371/journal.pone.0327503 (PMC12266389; doi:10.1371/journal.pone.0327503)

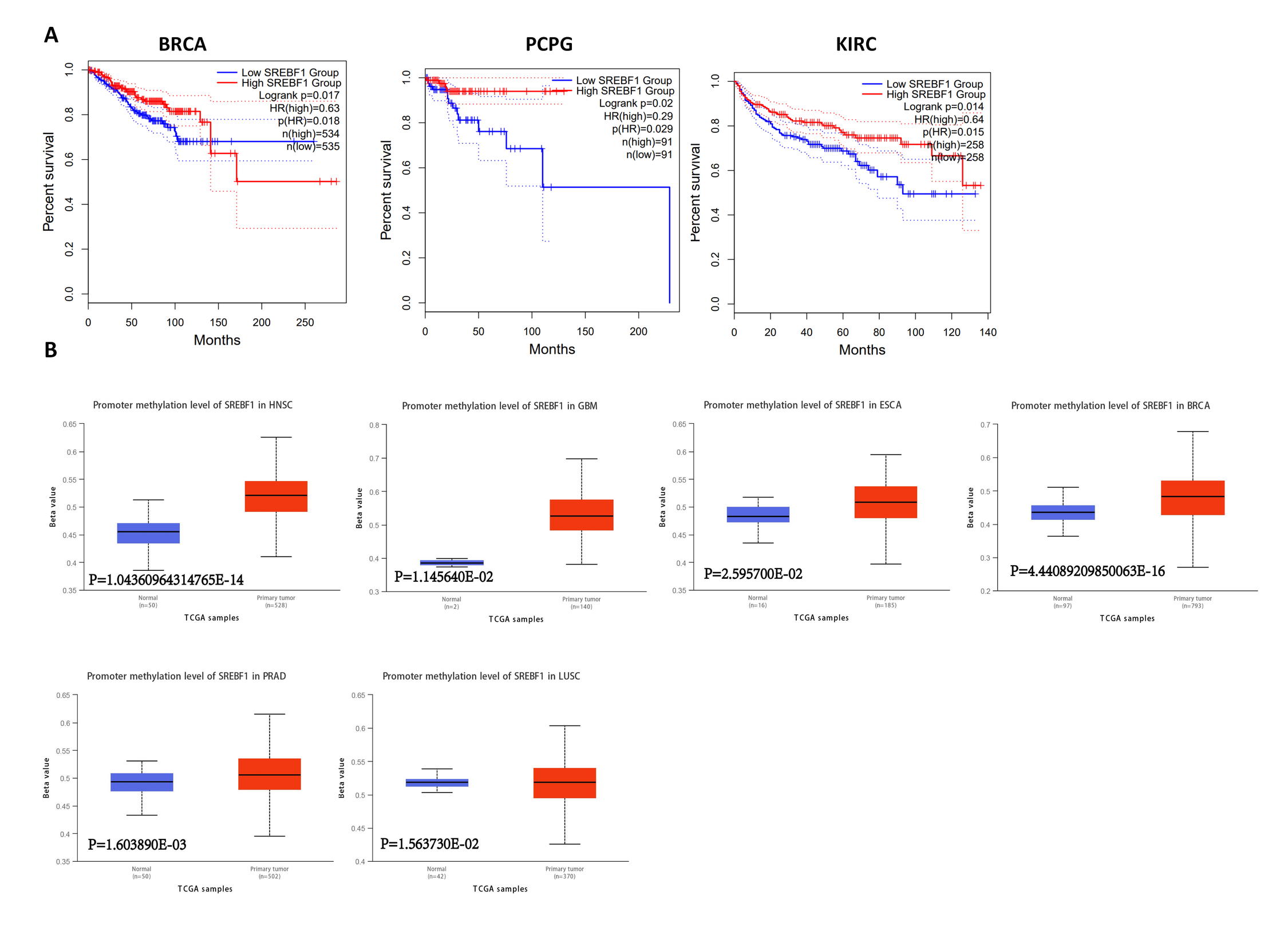

Supplement: S1 Fig — (B) The methylation values of SREBF1 between normal and primary tumor tissues. (TIF) [file pone.0327503.s001.tif]

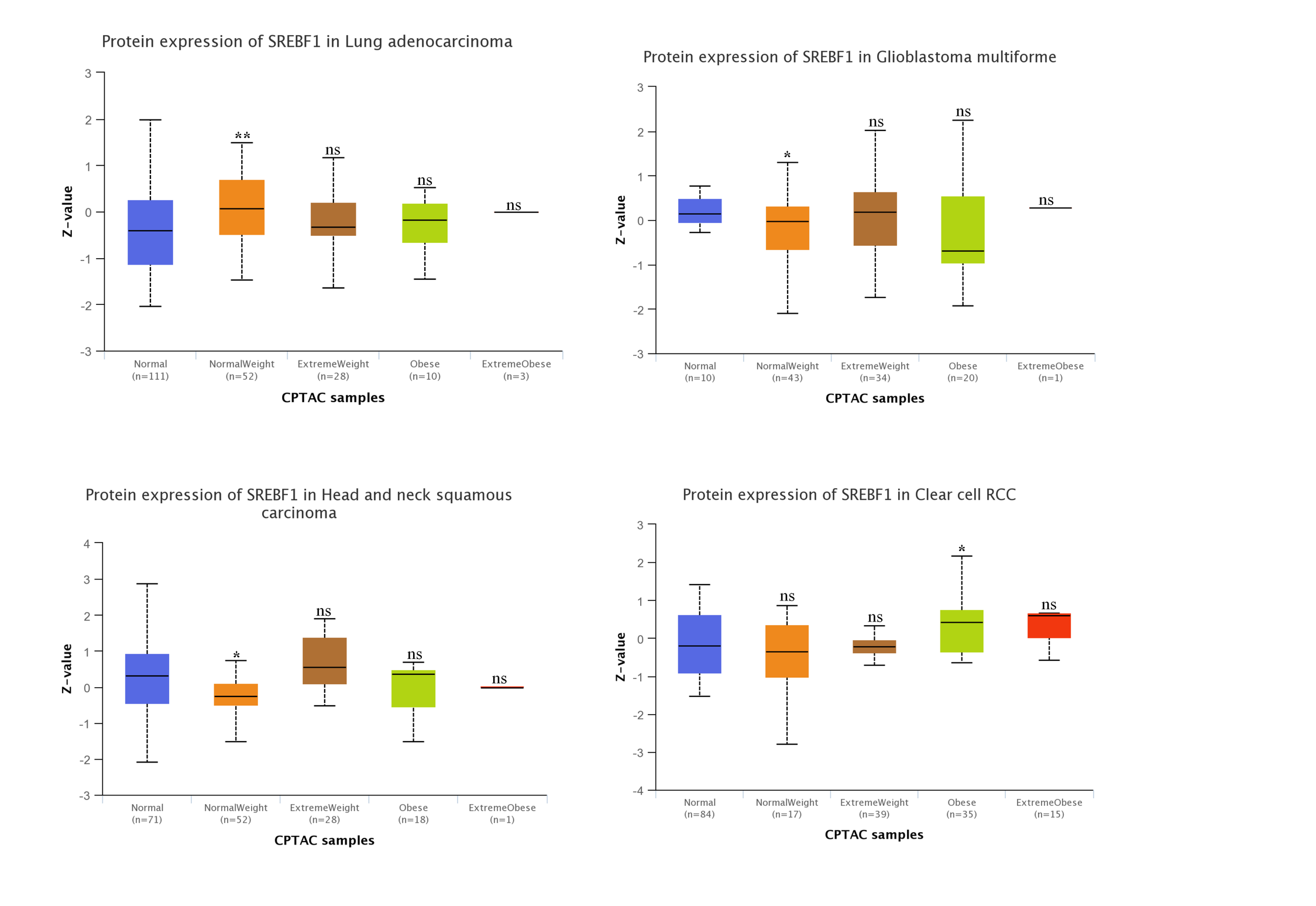

Supplement: S2 Fig — (TIF) [file pone.0327503.s002.tif]
